# Editorial: Special Issue on “New Frontiers in Molecular Mechanisms and Therapies in Neurological Diseases”

**DOI:** 10.3390/ijms24065601

**Published:** 2023-03-15

**Authors:** Ashu Johri

**Affiliations:** Independent Researcher, New York, NY 10021, USA; johri.ashu@gmail.com

## 1. Discussion

**Figure 1:**
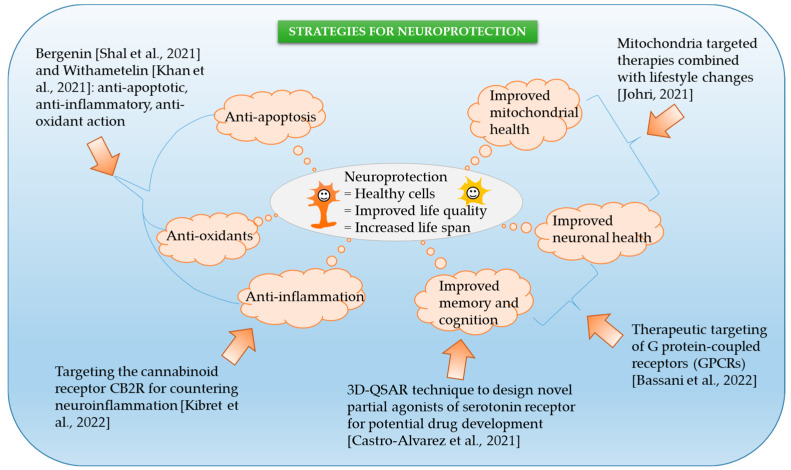
Outline of our SI, which includes a collection of studies that describe the various strategies employed for neuroprotection (Shal et al., 2021 [1], Khan et al., 2021 [2], Castro-Alvarez et al., 2021 [3], Johri, 2021 [4], Kibret et al., 2022 [5], Bassani et al., 2022 [6]).

We launched our Special Issue (SI) at the beginning of 2021, with the hope to bring together current research in the field of neurodegeneration. Our SI was welcomed by the world science community with enthusiasm, and we published six articles that focused on finding solutions to end the epidemic of neurodegenerative diseases (Figure 1). 

This editorial aims to highlight the studies published in this SI. In this series, Shal et al. reported on the neuroprotective effects of bergenin, a type of naturally occurring polyphenol compound, which is known to have antiulcerogenic, antiapoptotic, anti-inflammatory and antioxidant properties [1]. Using a 5x familial Alzheimer’s disease (AD) transgenic mouse model (5xFAD), the authors demonstrated that the bergenin (60 mg/kg) treatment prevented Aβ (1–42) aggregation in the hippocampus, significantly attenuated the memory deficit, and displayed the restoration of lipids, proteins and their derivatives [1]. Moreover, a concomitant increase in antioxidant levels, along with the suppression of pro-inflammatory cytokines, oxidative stress and nitric oxide production, was observed in specific brain regions isolated from the bergenin-treated 5xFAD mice.

A bidirectional correlation is suggested to exist between neurodegenerative diseases such as Alzheimer’s and Parkinson’s and chronic pain. In the next article of this SI, Khan et al. showed the efficacy of withametelin, a steroidal compound isolated from leaves of *Datura metel* L., in reducing neuropathic pain induced by vincristine in mice [2]. They showed that withametelin works by blocking the TRPV1 (transient receptor potential vanilloid 1)/TRPM8 (transient receptor potential melastatin 8) and P2Y nociceptors as well as MAPK (mitogen-activated protein kinase) signaling. Additionally, withametelin showed neuroprotective potential by reducing apoptosis, inflammatory cytokines, oxidative stress and genotoxicity in this pain model [2]. The ability of withametelin to inhibit the TRP nociceptors as well as all three MAPK pathways is especially impressive because this type of inhibition was previously shown to relieve inflammatory and neuropathic pain. 

The next study in this collection is by Castro-Alvarez et al., who employed the three-dimensional quantitative structure–activity relationship (3D-QSAR) technique to design novel partial agonists of the serotonin receptor (5-hydroxytryptamine receptor 4 (5-HT4R)) with potential biological activity [3]. 5-HT4R is highly expressed in the brain regions associated with memory and cognition and plays an important role in neurological diseases such as AD and PD. However, the development of 5-HT4R agonists is hampered by their inability to cross the blood–brain barrier and by their pharmacokinetic/pharmacodynamic behavior. To overcome these concerns, Castro-Alvarez et al. conducted a computational 3D-QSAR study of a dataset of molecules with agonist activity on 5-HT4Rs, narrowing these down to just a few compounds with the best predicted biologic activity [3]. They then designed novel agonists based on the most potent compounds and performed absorption, distribution, metabolism, excretion and toxicity (ADMET) predictions, as well as drug-likeness, for the newly designed compounds.

The next article in this series is a review where the focus was on AD as a multifactorial disease, for which mitochondrial dysfunction is at the forefront [4]. We discussed the mitochondrial dysfunction that takes place upstream of overt Aβ toxicity and disease phenotypes. Abnormal glucose metabolism and oxidative damage were also discussed, for which there is a broad base of evidence. More recently, health conditions related to lifestyle such as hypertension, stroke, diabetes and hypercholesterolemia have been considered as risk factors for AD. As a result of the multifactorial nature of the disease, therapies are also being tried from a variety of viewpoints. These include mitochondria-targeted therapies as well as lifestyle changes that could help restore mitochondrial health and the general overall health of an individual [4]. 

Amyotrophic lateral sclerosis (ALS, also called Lou Gehrig’s disease or motor neuron disease) is a devastating progressive neurodegenerative disease that affects nerve cells in the brain and spinal cord, causing a loss of control of muscle function. Bassani et al. compiled and described the progress made in recent years pertaining to the involvement of G protein-coupled receptors (GPCRs), a very important and widely studied class of biological entities, in the pathogenic process, as well as the potential of GPCRs as drug targets for ALS [5]. In the first part of their review, Bassani et al. summarized the existing small molecules currently used in U.S. Food and Drug Administration (FDA) clinical trials for ALS treatment, and in the latter part of the review, the authors discussed the evidence regarding the therapeutic potential of GPCRs for ALS treatment [5]. 

Two of the widely studied GPCRs include the cannabinoid receptors (CBRs) CB1R and CB2R. The therapeutic potential of these receptors in the context of neurodegenerative as well as neuropsychiatric disorders, including AD, PD, Huntington’s disease (HD), anxiety, depression, schizophrenia and addiction, was reviewed by Kibret et al. [6]. Cannabinoids produce anti-inflammatory actions via CB1R and CB2R and delay the progression of neuroinflammatory diseases. Both CB1R and CB2R are expressed in the brain. An important distinction is that compared to the CB1Rs, CB2Rs are associated with fewer adverse effects. CB2R expression and its upregulation has been associated with nervous disorders that are linked to underlying neuroinflammation [6]. 

## 2. Conclusions

Overall, this Special Issue was successful in bringing together studies that explored and provided up-to-date mechanistic insights into potential therapeutic compounds for some of the major neurodegenerative diseases. The reviews in this SI highlighted the current research on neurodegeneration. This SI has been a small step in the right direction to help and encourage the scientific and medical community to continue research in this field as we move toward a pathway for success in finding effective treatments for at least some of these terrible diseases.
